# Characterization of immunosuppression of genotype A2dB1 variant infectious bursal disease virus isolated in Malaysia using specific pathogen-free and commercial broiler chickens

**DOI:** 10.14202/vetworld.2025.799-807

**Published:** 2025-04-07

**Authors:** Paniz Zarghami Dastjerdi, Mohd Hair Bejo, Nor Yasmin Abd Rahaman, Abdullahi Abdullahi Raji, Roikhwan Soontravanich, Shen Rong Tai, Abdul Rahman Omar

**Affiliations:** 1 Laboratory of Vaccine and Biomolecules, Institute of Bioscience, Universiti Putra Malaysia, 43400 UPM Serdang, Selangor, Malaysia; 2 Department of Veterinary Pathology and Microbiology, Faculty of Veterinary Medicine, Universiti Putra Malaysia, 43400 UPM Serdang, Selangor, Malaysia; 3 Department of Veterinary Pathology, Faculty of Veterinary Medicine, Usmanu Danfodiyo University, 840212 Sokoto, Nigeria; 4 Regional Operating Unit South East Asia and South Korea, Boehringer Ingelheim Singapore Private Limited, 199555 Singapore; 5 Boehringer Ingelheim Sdn. Bhd. Level 23A, Mercu Aspire, No.3, Jalan Bangsar, KL Eco City, Kuala Lumpur 59200, Malaysia

**Keywords:** bursal atrophy, immunosuppression, Newcastle disease vaccination, novel variant infectious bursal disease virus, poultry health

## Abstract

**Background and Aim:**

Infectious bursal disease (IBD) is an immunosuppressive disease caused by the IBD virus (IBDV), which adversely affects poultry vaccination programs. The novel variant IBDV (nvIBDV) has recently emerged in various regions, including Malaysia, raising concerns about its immunosuppressive potential and impact on Newcastle disease (ND) vaccination. This study aimed to investigate the immunosuppressive effects of the Malaysian nvIBDV strain (UPM1432/2019) in specific-pathogen-free (SPF) and broiler chickens and evaluate its influence on ND vaccine efficacy.

**Materials and Methods:**

SPF chickens were orally infected with nvIBDV across three passage levels to study virus-induced clinical signs, lesions, and atrophy. Broiler chickens were vaccinated with live and killed ND vaccines and subsequently challenged with nvIBDV to measure ND antibody titers. The genotype of nvIBDV was characterized using sequence analysis of segments A and B. Bursal histopathology and statistical analyses were conducted to evaluate the virus’s immunosuppressive effects.

**Results:**

Infected SPF chickens displayed no clinical signs but showed significant bursal atrophy and lesions across all passages (p < 0.05). Broilers infected with nvIBDV exhibited no mortality or clinical signs; however, ND antibody titers significantly declined by 14 days post-challenge (1493.0 ± 746.1) compared with the unchallenged group (2975.7 ± 189.5; p < 0.01). Histopathological analysis revealed severe depletion of the bursal follicles, lymphoid cell aggregation, necrosis, and hemorrhage. Genotyping of nvIBDV identified as genotype A2dB1, consistent with strains from other regions.

**Conclusion:**

The Malaysian nvIBDV strain causes subclinical infections in SPF and broiler chickens, resulting in bursal atrophy and reduced ND vaccine-induced antibody titers. Silent spread and immunosuppressive effects present significant challenges to poultry health management and vaccination efficacy. Enhanced diagnostic and biosecurity measures are crucial for mitigating its impact.

## INTRODUCTION

Infectious bursal disease (IBD) (Gumboro disease) is an acute viral disease affecting young chickens. The clinical signs vary depending on the viral strain but generally include listlessness, diarrhea, ruffled feathers, and dehydration. While the morbidity rate is high and the mortality rate is typically low, very virulent (vv) strains can cause mortality rates exceeding 60% [1]. This virus can cause macroscopic and microscopic lesions in the bursa of Fabricius, harming the immune system where the virus infects and destroys B cells [1]. Hence, chickens infected with IBDV have poor vaccine-induced immunity and often succumb to secondary bacterial infection. There is also an indirect consequence of virus-induced changes in immune cell regulation, which mediates the regulation of various cytokines [2].

IBD is caused by the IBD Virus (IBDV), a single-shelled, non-enveloped, and double-stranded RNA virus belonging to the family *Birnaviridae* of the genus *Avibirnavirus* [3]. The genome of IBDV comprises segments A and B. Segment A contains two open reading frames encoding viral protein (VP)5 and a polyprotein, which cleaves into VP2, VP3, and VP4 [1]. VP2 is a viral capsid protein critical to virus pathogenicity and immunity. Segment B encodes the VP1 RNA-dependent RNA polymerase protein [3].

Conventionally, IBDV can be divided into several groups: classical virulent, antigenic variant, vv, and attenuated strains [4]. The virus can be classified into seven genogroups based on the *VP2* gene [5, 6]. However, this classification does not encompass all the emerging strains; hence, an improved scheme for IBDV genotyping was proposed [7, 8]. The genotypes of IBDV were characterized based on segments A and B, where IBDVs can be classified into nine “A” genogroups and five “B” genogroups, respectively. It was demonstrated that the genotypes A1B1, A2B1, A3B2, and A8B1 in the proposed scheme matched the phenotypes of classic, variant, vv, and attenuated IBDV that are traditionally characterized [7, 8].

Recently, novel variant IBDV (nvIBDV) was detected in commercial poultry flocks vaccinated against IBD in several countries, including China [9–11], Japan [12], South Korea [13], Malaysia [14], Egypt [15], and Argentina [16]. Sequence analysis of the *VP2* gene confirmed that nvIBDV closely resembles variant IBDV strains previously isolated in the USA, where they shared several key amino acid residues, including 222T, 249K, and 318D. In addition, the nvIBDVs were isolated from IBD-vaccinated flocks that exhibited a range of symptoms, from generally healthy birds with no symptoms to those showing mild signs of IBD, including weight loss, malaise, and secondary infections. This suggests that current IBD vaccination may not protect against novel variant IBD (nvIBD) infection completely [9, 17].

Previous studies have reported that nvIBDV induces subclinical signs and no mortality in specific-pathogen-free (SPF) and broiler chickens. However, infected birds have bursal lesions [17, 18]. The silent spread of nvIBDV may complicate the diagnosis and control of other diseases.

Despite extensive research on IBDV, the emergence of nvIBDV strains has introduced new challenges in poultry disease management. While previous studies have reported nvIBDV infections in several countries, including China, Japan, South Korea, and Egypt, limited data are available on its prevalence and impact in Malaysia. In addition, existing studies primarily focus on the genetic characterization and pathogenicity of nvIBDV, with insufficient investigation into its immunosuppressive effects, particularly in relation to vaccine efficacy. The potential interference of nvIBDV with Newcastle disease (ND) vaccination remains inadequately explored, raising concerns about compromised immunity in commercial poultry flocks. Furthermore, there is a lack of comprehensive data on histopathological changes associated with nvIBDV infection across multiple viral passages. Given the increasing reports of nvIBDV-related immunosuppression, a deeper understanding of its pathogenesis and interaction with poultry vaccination programs is essential for effective disease control strategies.

This study aims to evaluate the immunosuppressive effects of the Malaysian nvIBDV strain (UPM1432/2019) in SPF and broiler chickens. It also seeks to assess the impact of nvIBDV infection on ND vaccine-induced antibody titers in commercial broilers. In addition, the study aims to characterize the genetic composition of the nvIBDV strain based on sequence analysis of genome segments A and B. Furthermore, histopathological changes in the bursa of Fabricius following nvIBDV infection across different viral passages will be investigated. These objectives will provide valuable insights into the immunosuppressive mechanisms of nvIBDV and inform the development of improved vaccination strategies for poultry health management.

## MATERIALS AND METHODS

### Ethical approval

This study included two trials, and ethical approval was obtained from the Institutional Animal Care and Use Committee (IACUC) of Faculty of Veteri-nary Medicine (FVM) and Universiti Putra Malaysia (UPM). The first animal study was approved under Animal Use Proposal (AUP) number UPM/IACUC/AUP-R072/2022. The second animal study was approved under AUP number UPM/IACUC/AUP-R034/2021.

### Study period and location

The study had two different trials, the first trial was conducted from September 2022 to October 2022 and the second trial was from August 2023 to Septem-ber 2023 at the Animal research facilities of FVM of UPM.

### Viruses and vaccine

The nvIBDV strain UPM1432/2019 was previously isolated and identified in Malaysia from outbreaks in commercial broilers from Selangor in 2019 [14]. Live ND LaSota (Boehringer Ingelheim Animal Health, Gainesville, USA) and killed ND LaSota (Boehringer Ingelheim Animal Health, Guadalajara, Mexico) were used in this study.

### nvIBDV infection in SPF chickens

Eight-day-old SPF White Leghorn embryonated eggs were purchased from Malaysian Vaccine Pharmaceuticals and hatched at the Avian Laboratory Facility of the FVM, UPM. One-day-old SPF chickens (n = 40) were transferred to the Animal Research Facility, FVM, UPM. These birds were housed in separate rooms in the negative control and infected groups with regulated temperatures and ventilation in stainless steel cages. All chickens had access to feed and water *ad libitum*. At 3 weeks old, SPF birds were orally infected with 1 mL (15% w/v bursal homogenate virus stock) of the nvIBDV strain UPM1432/2019 [14]. The birds were monitored daily, and after 6 days of challenge, they were sacrificed. The bursal body weight (BBW) ratio was calculated, and the pathological changes were closely examined. Bursal tissues were harvested, homogenized (15% w/v), and clarified by centrifugation at 3,100 g for 5 min at 4°C (Eppendorf Centrifuge 5810R, Germany). The supernatants were filtered with a 0.45 μm filter (HmbG, Germany) and inoculated into 4 weeks of SPF chickens. The procedure was repeated, and the virus was inoculated into 5-week-old SPF birds. Saline-inoculated birds of the same age were used as negative controls.

### Virus titration

The bursal homogenate (15% w/v) from the third passage of the nvIBDV strain UPM1432/2019 was filtered and inoculated into 10-day-old embryonated SPF eggs for virus propagation through chorioallantoic membrane (CAM) inoculation. CAM tissues were harvested from the dead embryos, pooled, and stored as stock [14]. The EID_50_ of the stock was determined using Reed and Muench’s method in 10-day-old embryonated SPF eggs.

### nvIBDV infection in broiler chickens

Grade A healthy 1-day-old commercial broilers were purchased from a commercial broiler farm. The birds were randomly divided into the control group (n = 30) and the ND-vaccinated group (n = 30). These groups were housed in separate rooms with controlled temperature and ventilation in stainless steel cages. All chickens had access to feed and water *ad libitum*.

The ND-vaccinated group received vaccines on three occasions: on day 1 (live and killed vaccines), day 11, and day 21 (live vaccine only). Live vaccination was performed via eyedrop (0.1 mL/bird), whereas the killed vaccine was administered through subcutaneous injection (0.2 mL) as recommended by the manufacturer (Boehringer Ingelheim Animal Health, Ingelheim, Germany).

Serum samples were collected from day-old chicks before vaccination and weekly thereafter to monitor the seroconversion rate. On day 28, the ND-vaccinated group was divided into two groups: unchallenged and challenged with 10^6.7^ EID_50_ of nvIBDV strain UPM1432/2019 through the oral route. The birds were monitored daily for clinical signs. On days 7 and 14, post-challenged (pc), serum samples were collected, and postmortem examination was conducted.

### Determination of maternal IBDV antibody response

Maternally derived antibody (MDA) titers for IBDV were measured using an IBDV antibody kit (IDEXX, USA) following the manufacturer’s protocol.

### ND-specific antibody production

The ND antibody titer was measured using the ND virus antibody kit (IDEXX) according to the manufacturer’s instructions.

### Determination of the bursa–spleen ratios and bursa score

The body weights of the chicken, bursa, and spleen were measured using a digital scale (US Solid, USA). A postmortem was performed to determine any organ abnormalities. The bursal and splenic ratios were calculated using the formula shown below, as previously described by Sharma *et al*. [19].

BBW or SBW = Weight of the organ (g)/Body weight (g) × 1000.

Where BBW is the bursal body weight and SBW is the spleen body weight.

The bursa score was calculated using a bursa meter (Boehringer Ingelheim, Germany). The bursa meter is a ruler-shaped tool with holes of different sizes numbered 1–8 used to measure the bursa size, which shows the atrophy of the bursa [20].

### RNA extraction

RNA was extracted from the CAM samples using a Kylt kit (SAN Group Biotech, Germany) according to the manufacturer’s protocol. The concentrations and purity of the RNA samples were measured using a nanodrop Bio-Spectrophotometer (Eppendorf, Germany).

### Genotyping of IBDV

Genotypes of the nvIBDV strain UPM1432/2019 based on segments A and B (GenBank accession No. Seg A: MT505343, Seg B: MT505348.1) were characterized, as described previously by Wang *et al*. [7] and Islam *et al*. [8]. Briefly, the virus nucleotide sequences were aligned with IBDV strains representing different genotypes from GenBank (Supplementary Tables) using MEGA11.0.13 software (Pennsylvania State University, USA). The phylogenetic trees were constructed using the maximum-likelihood method with 1000 bootstrap replicates using MEGA11.0.13 software. Phylogenetic trees were constructed using the maximum-likelihood method with 1000 bootstrap replicates.

### Histopathology

The bursa tissue samples were processed using the standard histological procedure and were stained with hematoxylin and eosin for histopathological examination [21]. The bursa lesions were scored according to their degree of severity, as described previously Hair Bejo and Ng [22].

### Statistical analysis

All statistical analyses were conducted using Statistical Package for the Social Sciences version 23 (IBM Corp., Armonk, NY, USA) and GraphPad Prism version 10.2 (GraphPad Software, San Diego, CA, USA). Data were summarized as means and standard deviations (mean ± SD). One-way and two-way analysis of variance with Tukey’s *post hoc* test was used to compare differences among multiple groups, while an independent sample t-test was employed for comparisons between two groups.

The BBW and spleen body weight ratios, bursal lesion scores, and ND antibody titers were analyzed to assess group differences. Statistical significance was set at p < 0.05, with highly significant differences considered at p < 0.01. Data visualization was performed using bar graphs and tables to highlight key findings. The assumptions of normality and homoscedasticity were verified before conducting parametric tests.

Where applicable, non-parametric tests were considered for data that did not meet normality assumptions. The statistical analysis ensured a robust interpretation of the immunosuppressive effects of nvIBDV in both SPF and broiler chickens.

## RESULTS

Throughout the trial, the infected chickens did not exhibit any clinical signs. In addition, the body weight of the infected chickens was not significantly different from that of the control chickens, regardless of virus passage (p > 0.05). On postmortem examination at 6 days post-challenge, the infected chickens showed increasing bursal lesions with virus passages. The BBW ratios in the infected group were consistently lower than those in the control group across all passages. In addition, the bursal lesions worsened by the third passage, presenting as congestion, edema, hemorrhage, and atrophy. The bursal scores (3.20 ± 0.45) and bursal ratio (1.60 ± 0.38) of the chickens inoculated with nvIBDV passage 1 (P1) were significantly different compared with those of the control birds (4.30 ± 1.15) and bursal ratio (5.35 ± 2.55), respectively. A similar trend was also recorded when the chickens were inoculated with nvIBDV P2 and P3 (p = 0.03) and (p < 0.001), respectively (Table 1).

**Table 1:** Body weight, bursal score, and bursal ratio of SPF chickens with different passages into Malaysian nvIBDV.

Age of the birds (Virus passage)	Group	Body weight (Mean ± SD)	Bursa score (Mean ± SD)	Bursa ratio (Mean ± SD)
Three weeks old (P1)	Control (n = 5)	288.33 ± 24.01	4.33^b^ ± 1.15	5.35^b^ ± 2.55
	Infected (n = 5)	263.20 ± 20.58	3.20^a^ ± 0.45	1.60^a^ ± 0.38
Four weeks old (P2)	Control (n = 5)	429.00 ± 84.96	5.70^b^ ± 0.58	5.64^b^ ± 1.09
	Infected (n = 5)	423.20 ± 65.80	3.60^a^ ± 0.55	1.77^a^ ± 0.22
Five weeks old (P3)	Control (n = 5)	541.33 ± 99.48	6.17^b^ ± 0.76	5.09^b^ ± 1.52
	Infected (n = 15)	502.33 ± 63.67	4.00^a^ ± 0.59	1.90^a^ ± 0.35

a Denote significant differences with the negative control group,

b Denote significant differences with the infected group. SPF=Specific-pathogen-free, nvIBDV=Novel variant infectious bursal disease virus, SD=Standard deviation

**Figure 1:**
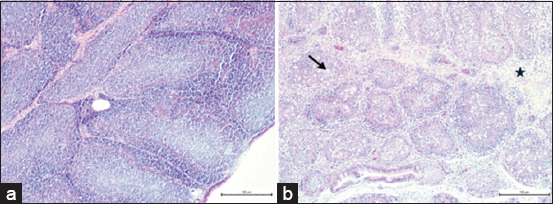
Histopathology of a bursa after third passage in (hematoxylin and eosin) 4×. (a) Negative control group and (b) infected group, severe depletion of the bursa (asterisk) and necrotic cells and cyst (arrow).

Histopathological examination of bursal tissues at P3 revealed severe depletion of bursal follicles, accompanied by lymphoid cell aggregations, necrotic cells, cyst formation, hyperemia, and hemorrhage. Thickened, corrugated, and vacuolated epithelium was also observed in some areas (Figure 1).

### Variant IBDV titration

The bursa homogenate from the third passage (15% w/v) was propagated in the SPF embryonated eggs (0.1 mL/egg) through CAM inoculation. The CAMs of the inoculated embryos that died were harvested and prepared as a stock of the challenge virus. The virus titer in EID_50_ was calculated using the Reed and Muench method: Proportionate Distance = ([Infectivity above 50%] − 50%)/([Infectivity above 50%] × [Infectivity below 50%]). The calculated titer was 10^6.4^ EID_50_/0.1 mL (Supplementary Table).

### Genotyping of IBDV

**Supplementary Figure 1:**
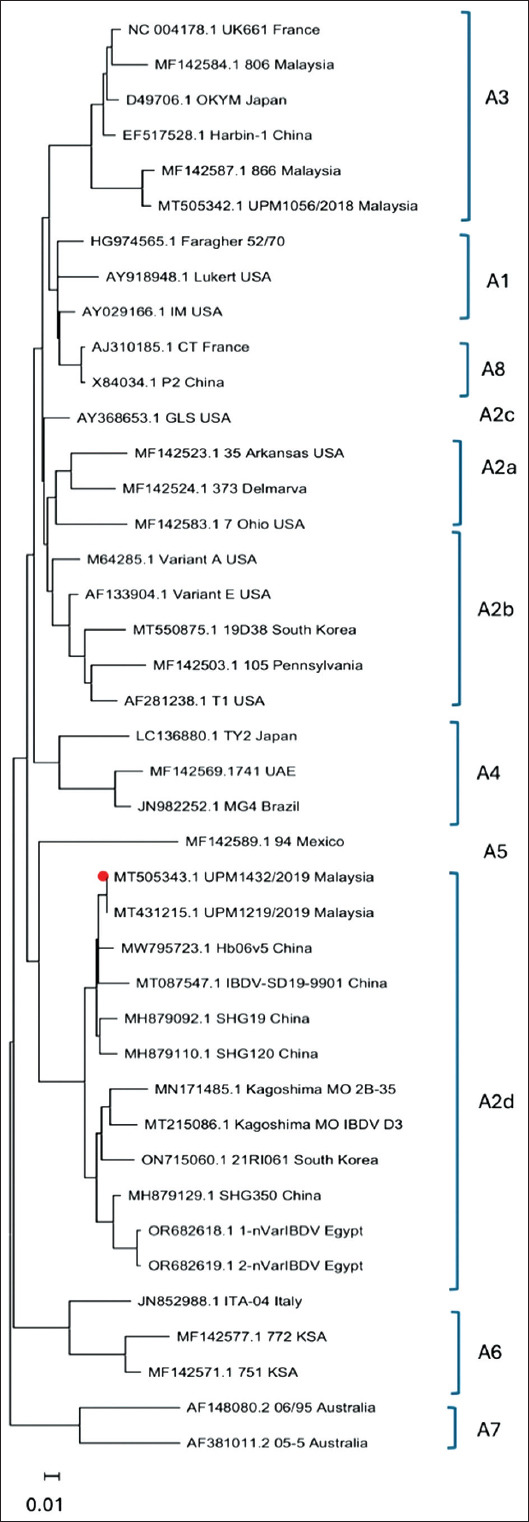
Phylogenetic analysis of the nucleotide sequences of segment A. The tree was generated using the maximum likelihood method with MEGA software.

**Supplementary Figure 2:**
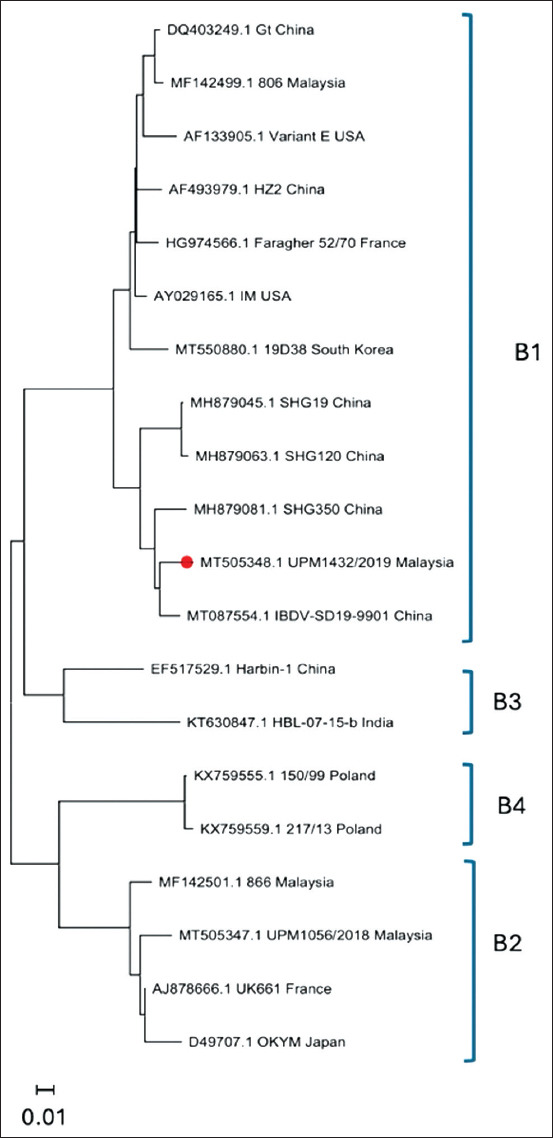
Phylogenetic analysis of the sequences of segment B. The tree was generated using the maximum likelihood method with MEGA software.

The genotyping of the nvIBDV strain UPM1432/2019 based on segments A and B confirmed that the virus belongs to the nvIBDV group, as genotype A2dB1, where segment A is of variant IBDV, while segment B is of non-vvIBDV groups, including the classical and antigenic variant viruses. The virus clustered together with some first nvIBDV isolates from China, Japan, and South Korea (Supplementary Figures 1 and 2).

### Identification of IBDV antibodies in broiler chickens

The MDA of day-old chicks against IBDV was randomly measured (n = 10), and the mean antibody titer was an average MDA of 2848.85 ± 1013.87.

### Detection of the ND antibody in broiler chickens

The mean MDA of day-old chicks against ND was high, 7337.8 ± 2021.5. The control groups showed a decrease in MDA, and by day 28, the ND antibody titer was below the cutoff value, indicating that the samples were negative for the ND antibody. However, chickens vaccinated with live and inactivated ND vaccines demonstrated a gradual increase in antibody titers ranging from 2714.3 ± 1710.1 to 2840.4 ± 1174.0 between the two groups before the IBD variant was challenged on day 28 (Table 2). The ND antibody titers between these two groups were not statistically significant (p = 0.66).

**Table 2:** Antibody responses against ND following IBD challenge in broiler chickens.

Group	ND antibody titer (Mean ± SD)				

	14 days	21 days	28 days	35 days (7 dpc)	42 days (14 dpc)
Negative control (n = 30)	1135.6^b^ ± 282.7	329.3^c^ ± 86.8	268.4^bc^ ± 78.7	196.8^bc^ ± 106.4	155.6^bc^ ± 15.9
ND-vaccinated and unchallenged (n = 10)	488.4^a^ ± 151.5	439.8^c^ ± 203.2	2840.4^a^ ± 1174.0	2860.8^a^ ± 737.0	2975.7^ac^ ± 189.5
ND-vaccinated and challenged (n = 20)	838.0 ± 325.5	998.4^ab^ ± 867.0	2714.3^a^ ± 1710.1	2309.1^a^ ± 1034.4	1493.0^bc^ ± 746.1

a Denote significant differences with the negative control group,

b Denote significant differences between vaccinated and non-challenged,

c Denote significant differences between vaccinated and challenged. IBD=Infectious bursal disease, ND=Newcastle disease, SD=Standard deviation

However, the ND titer showed a declining trend following the nvIBDV challenge. One week after the challenge, the mean antibody titer of the ND-vaccinated and challenged group decreased to 2309.1 ± 1034.3 compared with the ND-vaccinated and unchallenged group (2860.8 ± 737.0); however, the difference was not significant (p = 0.48). The decline continued, and at 2 weeks pc, a statistically significant drop in ND antibody titers was observed, from 2975.7 ± 189.5 in the ND-vaccinated and unchallenged to 1493.0 ± 746.1 in the ND-vaccinated and challenged group (p < 0.01) (Table 2).

### Variant IBDV infection in broiler chickens

No clinical signs were observed throughout the challenge study. The chicken weight did not differ significantly from the control group on day 35 (p = 0.26). Day 7 pc, the ND-vaccinated and challenged groups had significantly lower mean bursa ratios and bursa scores than the negative controls (p < 0.001). In addition, the spleen ratio was significantly higher in the group than in the negative control (p = 0.01) (Table 3).

**Table 3:** Mean bursal and spleen lesions in ND-vaccinated broiler chickens at 7 days post-challenge with variant IBDV.

Group	Body weight (Mean ± SD)	Bursa score (Mean ± SD)	Bursa ratio (Mean ± SD)	Spleen ratio (Mean ± SD)
Negative control (n = 10)	2300.11 ± 263.11	7.22^b^ ± 0.67	1.66^b^ ± 0.48	0.81^b^ ± 0.20
ND-vaccinated and challenged (n = 10)	2369.10 ± 185.57	3.90^a^ ± 0.56	0.60^a^ ± 0.13	1.19^a^ ± 0.29

a Denote significant differences with the negative control group,

b Denote significant differences with ND-vaccinated and challenged. IBDV=Infectious bursal disease virus, SD=Standard deviation, ND=Newcastle disease

A similar trend was observed on day 14 pc, when body weight between the ND-vaccinated and unchallenged groups was not significantly different (p = 0.77). The ND-vaccinated and challenged group had a significantly lower bursal ratio and score than the ND-vaccinated and unchallenged (p < 0.001). The spleen ratios were significantly different between the ND-vaccinated and unchallenged group and the ND-vaccinated and IBDV-challenged group (p < 0.001). However, the spleen ratios were not significantly different between the ND-vaccinated and unchallenged groups (p = 0.43) (Table 4).

**Table 4:** Mean bursal and spleen lesions in ND-vaccinated broiler chickens at 14 days post-challenge with variant IBDV.

Groups	Body weight (Mean ± SD)	Bursa score (Mean ± SD)	Bursa ratio (Mean ± SD)	Spleen ratio (Mean ± SD)
Negative control (n = 10)	2691.67 ± 373.42	7.33^c^ ± 0.71	1.98^c^ ± 0.47	1.20^b^ ± 0.97
ND-vaccinated and unchallenged (n = 10)	2841.40 ± 327.09	7.40^c^ ± 0.89	1.64^c^ ± 0.51	2.17^ac^ ± 0.43
ND-vaccinated and challenged (n = 10)	2878.73 ± 355.29	5.00^ab^ ± 0.47	0.50^ab^ ± 0.10	0.87^b^ ± 0.26

a Denote significant differences with the negative control group,

b Denote significant differences between the ND vaccinated and challenged,

c Denote significant differences with the ND vaccinated and challenge. IBDV=Infectious bursal disease virus, SD=Standard deviation, ND=Newcastle disease

## DISCUSSION

IBD is a highly contagious, acute, and immuno-suppressive disease that affects young chickens worldwide [1]. In Malaysia, IBD caused by vvIBDV was first reported in 1991 [23]. In addition, vvIBDV is a predominant IBDV strain in Europe and Asia [24]. The control of vvIBDV is achieved through farm sanitation practices, biosecurity, and vaccination.

The variant IBDV strain was first detected in the United States in the late 1980s [1]. Unlike the classical and vv strains of IBDV, the IBDV variant is antigenically distinct due to amino acid variations primarily at the hypervariable region of the *VP2* gene [24]. Chickens infected with IBDV variants isolated from the USA are characterized by no mortality and severe bursal depletion [25]. Recently, several Asian countries, including Malaysia, have reported the emergence of nvIBDV, which exhibits similar antigenic changes at the *VP2* gene [10, 14]. The first outbreak of this novel variant was reported in China [10]. Since then, the virus has been reported in several countries in Asia, including Japan and South Korea, and more recently in Egypt and Argentina, indicating the spreading potential of the virus worldwide; hence, it requires further research [11–16].

After the detection of nvIBDV in China, an improved scheme for IBDV genogroup classification was designed [7, 8]. Based on this classification, the nvIBDV strains were grouped into genogroup A2d for segment A. Since the first report of the outbreak of nvIBDV, various viruses have been reported from different countries, for example, the nvIBDV strains from China (SHG19, SHG20, and SHG350), South Korea (21RI061) and Japan (Kagoshima Mo D3) [10, 12, 13]. Sequencing and phylogenetic tree analysis of the nvIBDV strain UPM1432/2019 confirmed that the Malaysian nvIBD belongs to the same genogroup. In addition, segment B is clustered with the isolation of the nvIBDV strain from China from the B1 genogroup, which is a non-vvIBDV group, including the classical and antigenic variant viruses [7, 8]. Hence, nvIBDV, including UPM1943/2019, is classified as genotype A2dB1. Recently, a new reassortant strain of nvIBDV that has the same segment A features but segment B from the vv group, genotype A2dB3, has been detected in China [26]. At present, studies are underway to confirm the detection of reassortant IBDV strains in poultry farms in Malaysia and to determine their genomic sequences and biological properties in chickens.

No significant variations in body weight were detected among the chickens, although significantly lower mean bursa ratios and scores were recorded. Significant bursal depletion in nvIBD-infected chickens was observed with a reduction in ND antibody titers at 2309.1 ± 1034.4 and 1493.0 ± 746.1 at 7 and 14 dpc, respectively, confirming that the nvIBD can suppress ND vaccination. However, further studies are required to determine whether the lowering of the ND antibody titer following nvIBDV infection is associated with the inability to confer full protection against challenges with velogenic ND.

A study by Fan *et al*. [18] on the suppressive effect of nvIBDV on ND vaccination in broiler chickens in China showed that nvIBDV infection damages the bursa and spleen and interferes with ND vaccination, which is consistent with the results of the present study. Another study by *et al*. [27] on nvIBDV isolated from broiler chickens in Japan reported bursal atrophy, reduced BBW ratio in 28-day-old broilers, and induced immunosuppression by influencing factors on bursal atrophy and BBW ratio in the broilers; however, vaccination profiles were not studied. This is the first study on the immunosuppressive effect of Malaysian nvIBDV. Our findings confirmed that nvIBDV plays a crucial role in modulating humoral immunity following ND vaccination. This study indicates that it is vital to use a comprehensive set of investigation tools, such as molecular techniques such as polymerase chain reaction, sequencing, and serological techniques, such as enzyme-linked immunosorbent assay, to conduct meaningful field monitoring, diagnostic surveillance, and control measures of IBD [19, 28, 29].

## CONCLUSION

This study demonstrates the immunosuppressive effects of the Malaysian nvIBDV strain (UPM1432/2019) in SPF and broiler chickens. The findings indicate that while nvIBDV infection did not result in overt clinical signs, it led to significant bursal atrophy and histopathological lesions across multiple viral passages in SPF chickens (p < 0.05). In broiler chickens, nvIBDV challenge resulted in a substantial reduction in ND vaccine-induced antibody titers, particularly on 14 days pc (p < 0.01), suggesting its potential interference with vaccination efficacy. Histopathological analysis revealed severe bursal depletion, including lymphoid aggregation, necrosis, and hemorrhage. Furthermore, genotypic characterization confirmed that the Malaysian nvIBDV strain belongs to the A2dB1 genotype, aligning with nvIBDV isolates from other regions.

One of the key strengths of this study lies in its comprehensive approach, which integrates virological, immunological, and histopathological assessments to elucidate the impact of nvIBDV on poultry immunity. The use of controlled experimental conditions allowed for precise evaluation of virus-induced immunosuppression and vaccine interference. Moreover, the phylogenetic characterization of the Malaysian nvIBDV strain contributes valuable data to the global understanding of nvIBDV genetic diversity and evolution.

Despite its strengths, this study has certain limitations. The findings are based on controlled laboratory settings, which may not fully replicate field conditions where multiple environmental and management factors influence disease dynamics. In addition, the study focused on a single viral strain, and the long-term effects of nvIBDV infection on poultry productivity and susceptibility to secondary infections were not assessed. Further research is needed to explore the potential genetic reassortment of nvIBDV with other circulating strains and its implications for poultry disease management.

Future studies should focus on evaluating the impact of nvIBDV infection under commercial farming conditions and assessing its interactions with other pathogens. Long-term investigations into its economic consequences on broiler production and immune competence are essential. In addition, research into the development of novel vaccines and enhanced diagnostic tools is crucial to mitigate the immunosuppressive effects of nvIBDV. Strengthening biosecurity measures and implementing routine surveillance programs will be critical in preventing the silent spread of nvIBDV and ensuring effective poultry health management.

## DATA AVAILABILITY

The supplementary data can be available from the corresponding author on a reasonable request.

## AUTHOR CONTRIBUTIONS

PZD, MHB, NYAR, ARO, RS, AAR, and SRT: Conceptualization. PZD, SRT, AAR, and ARO: Methodology. ARO, RS: Project administration. ARO, MHB, NYAR: Supervision. PZD: Writing – original draft. PZD, MHB, NYAR, AAR, RS, SRT, and ARO: Writing – review and editing. All authors have read and approved the final manuscript.
